# Assessing Delay Patterns in Diagnosis and Care Among Breast Cancer Patients in Ethiopia

**DOI:** 10.1002/cnr2.70593

**Published:** 2026-05-31

**Authors:** Meklit Solomon Gebremariam, Segni Kejela, Yeabtsega Amlaku Asres, Erko Chala Beyene, Tsegab Alemayehu Bukate, Misgana Muluneh Ojamo, Kalsidagn Girma Asfaw, Meti Temesgen Negassa, Abenezer Tarekegne Legesse

**Affiliations:** ^1^ College of Health Sciences Addis Ababa University Addis Ababa Ethiopia; ^2^ Department of Surgery, College of Health Sciences Addis Ababa University Addis Ababa Ethiopia; ^3^ Saint Paul's Hospital Millenium Medical College Addis Ababa Ethiopia; ^4^ Armauer Hansen Research Institute Addis Ababa Ethiopia; ^5^ Department of Emergency Medicine, College of Health Sciences Addis Ababa University Addis Ababa Ethiopia; ^6^ Johns Hopkins University Baltimore Maryland USA; ^7^ MCM Comprehensive Specialized Hospital Addis Ababa Ethiopia; ^8^ Maastricht University Maastricht the Netherlands

**Keywords:** low‐and‐middle income countries, primary delay, provider delay, secondary delay, tertiary delay

## Abstract

**Background:**

In Ethiopia, breast cancer patients often present with advanced‐stage disease, leading to high mortality. This study aimed to quantify diagnostic and treatment intervals and identify their association with stage at presentation.

**Aim:**

The aim of this study is to determine the role of specific types of delay on the stage of disease presentation for breast cancer.

**Methods and Results:**

A multicenter cross‐sectional study was conducted at Ethiopia's two largest tertiary oncology centers. Data from 205 histologically confirmed breast cancer patients were analyzed using multivariable logistic regression and random forest machine learning to evaluate the impact of various time intervals on disease stage.

The median total delay was 5.05 months, with more than half of patients exceeding a six‐month interval from symptom onset to treatment initiation. Primary delay (patient interval) was the dominant contributor, accounting for over 77% of the total variance in delay. Total delay > 6 months was independently associated with a 4.26‐fold higher odds of advanced‐stage (Stage III/IV) presentation (AOR 4.26, 95% CI: 2.07–8.77, *p* < 0.001). Lack of awareness was the primary reason for delay (77.8%), while complementary medicine use and absence of pain were not significantly associated with prolonged total delay.

**Conclusion:**

The majority of delays occur during the pre‐clinical phase. Reducing primary delay through targeted community awareness and improved symptom recognition is critical to achieving earlier diagnosis and reducing breast cancer mortality in Ethiopia.

## 1 Introduction

Breast cancer survival has improved dramatically in high‐income countries, where 5‐year survival exceeds 80%, largely due to organized screening programs and timely initiation of treatment. In contrast, low‐ and middle‐income countries (LMICs), particularly in sub‐Saharan Africa (SSA), continue to experience disproportionately high mortality relative to incidence [1, 2]. In Ethiopia, breast cancer is the most common malignancy among women and a leading cause of cancer‐related death [3]. A substantial proportion of patients present with advanced‐stage disease, severely limiting curative treatment options and contributing to poor survival outcomes [4].

Accumulating evidence suggests that prolonged diagnostic and treatment intervals, rather than intrinsic biological aggressiveness alone, play a central role in late‐stage presentation in SSA [5]. The patient pathway from symptom recognition to initiation of definitive therapy consists of multiple sequential intervals, including the patient interval (symptom onset to first healthcare contact), the diagnostic interval (first contact to histologic confirmation), and the treatment interval (diagnosis to treatment initiation) [6]. These intervals are shaped by complex interactions between health literacy, sociocultural beliefs, economic constraints, and health system capacity. However, the relative contribution of each component interval to overall delay and stage at diagnosis remains incompletely characterized in the Ethiopian context [6].

Understanding the temporal structure of breast cancer care is essential for informing targeted interventions aligned with the World Health Organization Global Breast Cancer Initiative, which emphasizes timely diagnosis and treatment as a cornerstone of mortality reduction [7]. In particular, identifying which phase of the diagnostic pathway most strongly predicts advanced‐stage disease could guide resource allocation toward patient‐directed awareness interventions, system‐level reforms, or both.

The present multicenter study therefore aimed to (1) quantify diagnostic and treatment time intervals among patients with breast cancer in Ethiopia, (2) identify determinants of prolonged delay, and (3) evaluate the independent association between these intervals and stage at presentation using both multivariable regression and machine learning approaches. By reconstructing the diagnostic journey of patients receiving care at the country's two largest tertiary oncology centers, this study seeks to clarify the temporal drivers of advanced‐stage presentation and provide evidence to inform national cancer control strategies.

## 2 Methods

### 2.1 Study Design

This was a multicenter cross‐sectional analytical study with retrospective reconstruction of diagnostic and treatment time intervals among patients with histologically confirmed breast cancer. Although structured interviews were scheduled and administered as participants were enrolled, the resulting assessment of diagnostic pathway intervals was essentially a reconstruction based on retrospective patient recall of symptom onset and healthcare encounters. The primary objective was to quantify diagnostic and treatment time intervals and evaluate their association with stage at presentation.

### 2.2 Setting

The study was conducted at Tikur Anbessa Specialized Hospital (TASH) and Saint Paul's Millennium Medical College (SPMMC), the two largest tertiary referral centers providing comprehensive oncology services in Addis Ababa, Ethiopia. Both institutions serve as national referral centers and provide surgical oncology, medical oncology, radiotherapy, pathology, and imaging services for breast cancer patients. Data were collected continuously between June 2021 and August 2023. Recruitment was conducted consecutively among eligible patients attending surgical or oncology outpatient follow‐up clinics during this period.

### 2.3 Participants

All patients presenting during the study period were screened for eligibility. Inclusion criteria were histologically confirmed breast cancer and initiation of definitive treatment (surgery, chemotherapy, radiotherapy, or hormonal therapy) within 6 months prior to enrollment. The 6‐month criterion was applied to minimize recall bias in reconstructing diagnostic intervals. Exclusion criteria included recurrent breast cancer, initiation of treatment more than 6 months before enrollment, incomplete staging data, incomplete interviews, and absence of retrievable medical records. Of 217 patients screened, 205 met eligibility criteria and were included in the final analysis (Figure S1).

We acknowledge that restricting inclusion to patients who initiated treatment within 6 months prior to enrollment may have excluded individuals with extremely prolonged diagnostic pathways or those who never reached tertiary care. This may underestimate the true duration of diagnostic intervals at the population level.

### 2.4 Variables

The primary outcome was stage at diagnosis, classified according to the American Joint Committee on Cancer (AJCC) 8th edition staging system. Stage was dichotomized into early stage (Stage I–II) and advanced stage (Stage III–IV) for regression analyses.

The primary exposures were diagnostic and treatment time intervals measured in months. Details of which are described in the operational definition section. For comparability with prior literature and to explore health‐system components, individual sub‐intervals were also analyzed separately. Total interval was additionally categorized as ≤ 6 months versus > 6 months based on clinically relevant thresholds reported in global oncology literature involving over 100 000 breast cancer patients [8].

Additional covariates included age, sex, region of residence (urban versus non‐urban), educational level, marital status, family history of breast cancer, initial presenting symptom (including presence or absence of breast pain), comorbidities, and utilization of complementary and alternative medicine (CAM).

### 2.5 Operational Definitions and Ascertainment of Diagnostic and Treatment Delays

To reconstruct the diagnostic and treatment pathway, we disaggregated the patient journey into sequential, non‐overlapping temporal intervals based on established frameworks in global oncology. Each interval was operationally defined a priori and quantified in months. All intervals were reconstructed using a combination of structured patient interviews and medical record verification. Dates were recorded as precisely as possible (day/month/year), and intervals were calculated by subtracting earlier dates from subsequent milestone dates. When exact dates were not recalled, patients were encouraged to approximate timing relative to memorable personal, cultural, or national events; the 15th of the reported month was assigned when only the month was recalled. The categorization of delay intervals was informed by the recommendations of the Aarhus Statement for studies on early cancer diagnosis and represents a contextual refinement of this framework adapted to the Ethiopian healthcare setting [9]. Because the Aarhus Statement fails to incorporate patients' decisions to seek care, an element we consider vital, we sought additional models to study delay more comprehensively [9]. Danna and colleagues proposed a model that incorporates the decision to seek care as a ‘primary delay’ in resource‐limited settings; although this was originally applied to a different disease, it was used here in conjunction with the Aarhus Statement [10].

### 2.6 Primary Delay (PD)

Primary delay was defined as the time interval between initial symptom onset and the patient's decision to seek care at a healthcare facility.

Symptom onset was defined as the date when the patient first noticed a persistent breast‐related abnormality (e.g., lump, swelling, nipple discharge, skin change). The “decision date” was defined as the time when the patient internally resolved to seek medical evaluation, regardless of whether the visit occurred immediately.

This interval was obtained exclusively through structured interview, as decision‐making events are not documented in medical records. To enhance recall validity, participants were asked to describe the circumstances surrounding symptom recognition and the cognitive process leading to care‐seeking. Chronological anchoring techniques were used to link the decision to specific life events (religious holidays, agricultural seasons, family events, or national holidays).

Primary delay captures the appraisal and help‐seeking phase and reflects health literacy, symptom interpretation, sociocultural beliefs, fear, stigma, and competing responsibilities.

### 2.7 Secondary Delay (SD)

Secondary delay was defined as the time interval between the patient's decision to seek care and the first actual visit to any healthcare provider (public, private, or traditional biomedical facility).

The first healthcare contact was defined as the first in‐person consultation with a licensed healthcare provider (health center, clinic, hospital). Visits to religious institutions or traditional healers were not classified as healthcare contact unless they occurred within a formal biomedical setting.

The date of first healthcare contact was primarily self‐reported and cross‐verified when possible with referral slips or outpatient records. In cases where discrepancies occurred, documented clinical dates were prioritized.

Secondary delay reflects logistical barriers between intention and action, including financial constraints, transportation limitations, household responsibilities, or healthcare access barriers.

### 2.8 Provider‐Associated Delay (PAD)

Provider‐associated delay was defined as the interval between the patient's first healthcare visit and referral to a definitive cancer treatment center, attributable to healthcare system factors.

Operationally, PAD was identified when one or more of the following occurred:

- The patient was reassured that the condition was benign without appropriate diagnostic confirmation (e.g., no imaging, biopsy, or referral).
- Empirical treatment was initiated for presumed benign conditions (e.g., mastitis, abscess) without malignancy exclusion.
- The provider advised observation or delayed follow‐up without appropriate diagnostic workup.
- The provider expressed reluctance to refer to a tertiary oncology center despite persistent symptoms.

Ascertainment of PAD relied on patient report of the content of the diagnostic discussion during early encounters, combined with review of referral documentation and medical records when available. Patients were specifically asked whether malignancy was discussed, whether diagnostic tests were performed, and whether they were advised that the lesion was “not cancer” prior to histological confirmation.

The start date of PAD was the first healthcare visit date. The end date was the documented referral date to a definitive care center (TASH or SPMMC). If referral occurred during the first visit, PAD was recorded as zero.

PAD reflects diagnostic error, delayed suspicion, limited provider awareness, resource limitations, and referral inefficiencies.

### 2.9 Referral Delay

Referral delay was defined as the time interval between formal referral from a primary or secondary healthcare facility and the patient's arrival at a definitive tertiary oncology center.

The referral date was verified through referral letters, stamps, or documented transfer notes. The arrival date was confirmed using hospital registration records at TASH or SPMMC.

Referral delay reflects geographic barriers, transportation limitations, appointment scheduling delays, administrative bottlenecks, and patient‐level logistical constraints during inter‐facility transfer.

### 2.10 Tertiary Delay (TrD)

Tertiary delay was defined as the time interval between histological confirmation of breast cancer diagnosis and initiation of the first definitive cancer treatment (surgery, chemotherapy, radiotherapy, or hormonal therapy).

The diagnosis date was defined as the biopsy confirmation date documented in pathology reports. The treatment initiation date was verified using operative records, chemotherapy initiation sheets, radiotherapy logs, or pharmacy records.

TrD captures health system efficiency at the tertiary level, including waiting times for surgery, pathology processing, treatment scheduling, multidisciplinary coordination, and resource availability.

### 2.11 Total Delay

Overall delay was defined as the cumulative time interval from initial symptom onset to initiation of definitive cancer treatment.

Mathematically:

Total Delay=PD+SD+PAD+Referral Delay+TrD

### 2.12 Data Sources and Measurement

Data were collected using a structured interviewer‐administered questionnaire. Interviews were conducted entirely in participants' native languages by trained medical personnel. To improve recall accuracy, chronological anchoring techniques were used, linking symptom onset and healthcare encounters to memorable personal, religious, or national events.

Medical records were accessed to verify key clinical dates, including date of biopsy confirmation, date of first consultation at the tertiary center, date of treatment initiation, histopathological diagnosis, and AJCC stage at diagnosis. When discrepancies arose between self‐reported dates and documented clinical records, medical record dates were prioritized. Despite these measures, some degree of recall bias cannot be excluded, particularly for early symptom onset.

CAM use was assessed by asking patients whether they sought religious healing (e.g., prayer, holy water), traditional healers, herbal remedies, or combined modalities during any interval of their diagnostic pathway.

### 2.13 Bias

Several measures were implemented to minimize bias. Consecutive recruitment reduced selection bias within participating institutions. Restricting enrollment to patients who initiated treatment within 6 months prior to interview minimized long‐term recall bias. Medical record verification improved temporal accuracy of key clinical milestones. Multivariable regression modeling was used to adjust for potential confounders.

Nonetheless, potential sources of bias remain. The study population was limited to patients who successfully reached tertiary referral centers and initiated treatment; therefore, women who never accessed tertiary care or died before treatment initiation were not captured. This introduces potential selection and survivor bias. Additionally, despite structured interviewing techniques, retrospective recall of symptom onset may be imprecise.

### 2.14 Study Size

Sample size was calculated using a double population proportion formula based on preliminary data from 30 patients, which indicated that approximately 57% experienced delays greater than 6 months. Assuming 80% power and a two‐sided alpha of 0.05, the minimum required sample size was 197 patients. A total of 205 eligible patients were included in the final analysis.

### 2.15 Quantitative Variables

Time intervals were treated as continuous variables measured in months. Because interval distributions were positively skewed, medians and interquartile ranges (IQR) were used as primary descriptive measures. Means and standard deviations were additionally reported to allow comparison with prior studies. For regression analyses, delay variables were modeled both continuously (per one standard deviation increase) and categorically (> 6 months vs. ≤ 6 months).

### 2.16 Statistical Analysis

All statistical analyses were performed using IBM SPSS Statistics for Windows, Version 24 (IBM Corp., Armonk, NY, USA) and Python (version 3.12; Python Software Foundation, Wilmington, DE, USA). Descriptive and inferential statistical analyses were conducted in SPSS. Descriptive statistics included frequencies and percentages for categorical variables and medians with interquartile ranges (IQR) for skewed continuous variables. Group comparisons were performed using the Mann–Whitney *U* test or Kruskal–Wallis test for non‐normally distributed variables and chi‐square tests for categorical variables.

The primary analytic approach used binary logistic regression to assess associations between diagnostic intervals and advanced stage at presentation. Variables were selected for multivariable modeling based on clinical relevance, established literature, and univariable association with *p* < 0.20. A hierarchical adjustment strategy was employed: initial models included delay variables alone, followed by sequential adjustment for demographic variables (age, sex), socioeconomic variables (education, residence), and clinical characteristics.

Given the mathematical dependency between total interval and its component sub‐intervals, these variables were not entered simultaneously into the same regression model. Multicollinearity was assessed using variance inflation factors and correlation matrices. Because the primary interval demonstrated a strong correlation with total interval (*r* = 0.88), separate models were constructed for total interval and individual components.

Machine learning analyses, including random forest classification and variable importance estimation, were conducted using Python (version 3.12) with the scikit‐learn library (version 1.8.0). Model performance was evaluated using overall classification accuracy, and variable importance was quantified using mean decrease in Gini impurity.

All tests were two‐sided, and statistical significance was defined as *p* < 0.05.

### 2.17 Ethical Considerations

Ethical approval was obtained from the Institutional Review Board of the Department of Surgery at Addis Ababa University College of Health Sciences and Saint Paul's Millenium Medical College through an expedited review process for a residency graduation thesis Written informed consent was obtained from all participants prior to enrollment. All data were anonymized and handled in accordance with institutional and national ethical guidelines.

## 3 Results

Of the 217 patients approached, 205 (94.5%) met eligibility criteria and were included in the final analysis. Eleven patients were excluded: 6 due to incomplete staging data and 5 who did not complete the interview process. The median age at presentation was 41 years (mean 43.7 ± 12.5 years), with nearly half of the cohort under 40 years of age. Male patients comprised 4.4% (*n* = 9) of the study population. Additional socio‐demographic characteristics are summarized in Table S1.

**FIGURE 1:**
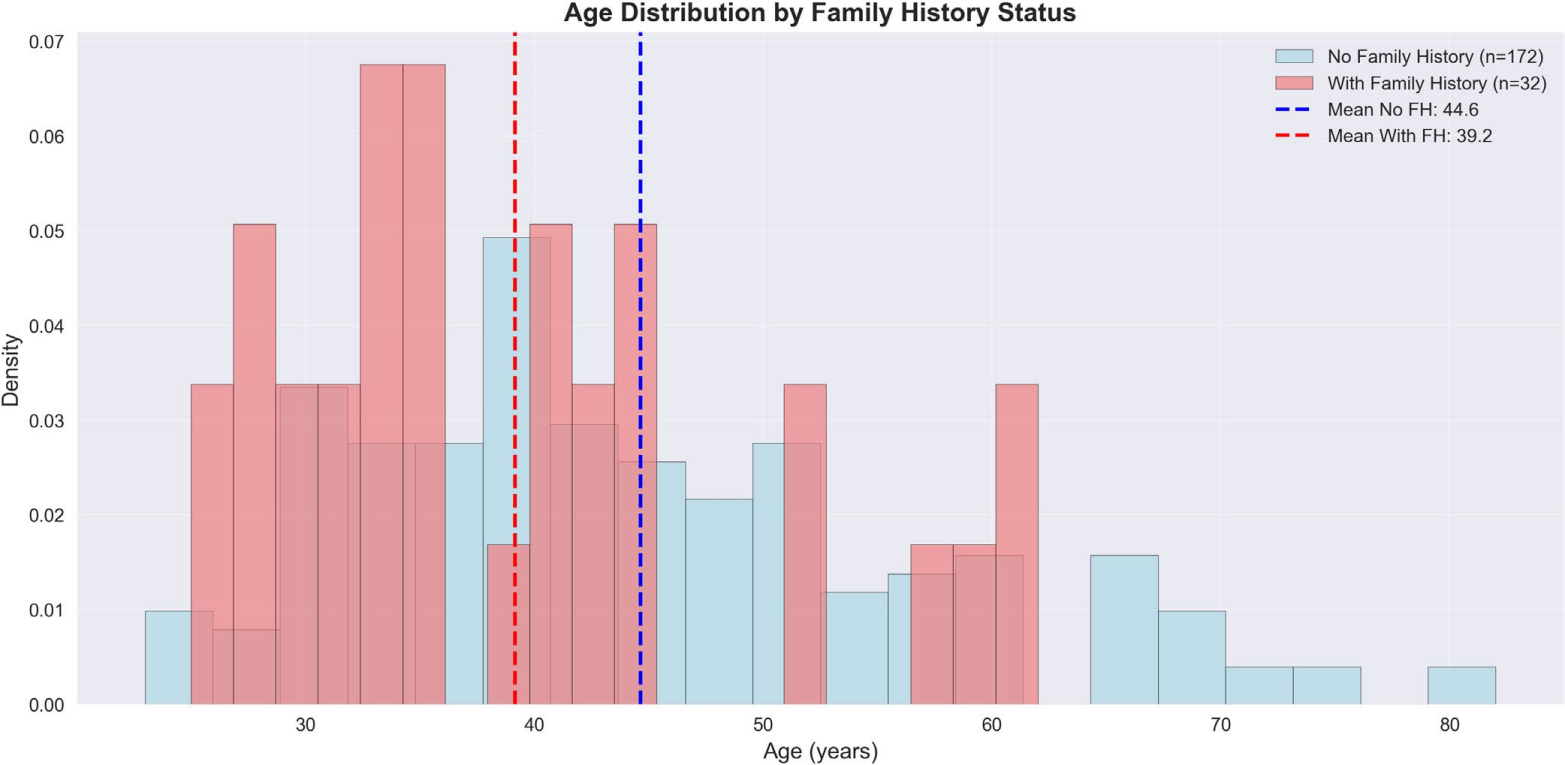
Comparison of age distribution between respondents with and without family history of breast cancer.

A family history of breast cancer was reported in 15.5% of patients (*n* = 32). Patients with a positive family history (FH) presented at a younger age than those without such history, with a median age of 36 years (mean 40.4 ± 12.3) compared to 42 years (mean 45.0 ± 13.1), respectively (Mann–Whitney *U* test, *p* = 0.019), Figure 1.

A breast lump was the most frequently reported initial symptom, accounting for 88.3% (*n* = 181) of cases, followed by breast pain and axillary swelling. Regarding clinical stage at diagnosis, Stage III was the most common, found in 46.8% (*n* = 96) of patients, closely followed by Stage II in 30.2% (*n* = 62). Stages I and IV were the least common, reported in 6.3% (*n* = 13) and 16.6% (*n* = 34) of cases, respectively. Additional information on stage, grade, histologic types, and comorbidities is provided in Table S2.

The median total delay (TD) was 5.05 months (mean 6.6 ± 9.87 months), reflecting a right‐skewed distribution. The median primary delay was 3 months (mean 1.78 ± 2.75 months), while the median tertiary delay was 1 month Table 1.

**TABLE 1:** Descriptive statistics of delay types across the diagnostic pathway.

Delay type	Median	Mean	SD^a^	Minimum	Maximum	IQR^b^
Primary delay	3.0	6.6	9.87	0.0	84.0	6.0
Secondary delay	0.0	0.38	0.83	0.0	6.0	0.5
Referral delay	0.0	0.83	5.63	0.0	78.0	0.25
Provider delay	0.0	2.45	7.42	0.0	48.0	1.5
Diagnostic delay	4.0	8.87	12.19	0.0	84.35	10.57
Tertiary delay	1.0	1.78	2.75	0.0	15.0	1.0
Total delay	5.05	9.8	12.1	0.0	86.35	10.75

^a^ Standard deviation.

^b^ Inter‐quartile range.

The majority of patients (77.8%, *n* = 161) attributed their primary delay to lack of awareness regarding breast cancer symptoms. Additionally, 30.9% (*n* = 64) reported the absence of pain as a reason for delaying the decision to seek medical care. Financial constraints were infrequently cited, with only 3.9% (*n* = 8) identifying lack of monetary support as a contributing factor. A comparable proportion of patients reported lack of time as a reason for postponing the decision to present to a healthcare facility.

**FIGURE 2:**
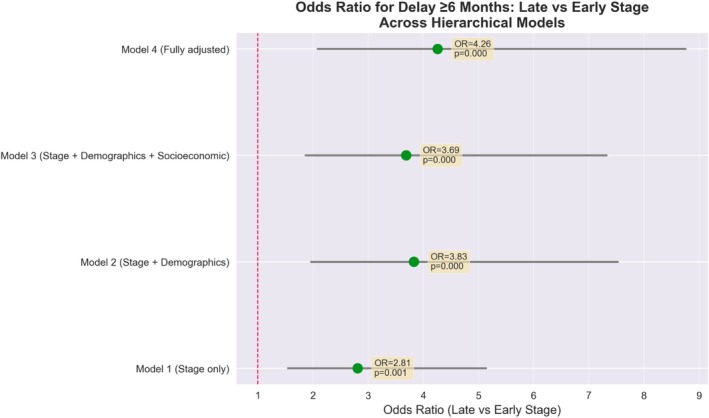
Association between disease stage and selected outcomes: Multivariable logistic regression models with progressive adjustment for demographic and sociodemographic factors.

**FIGURE 3:**
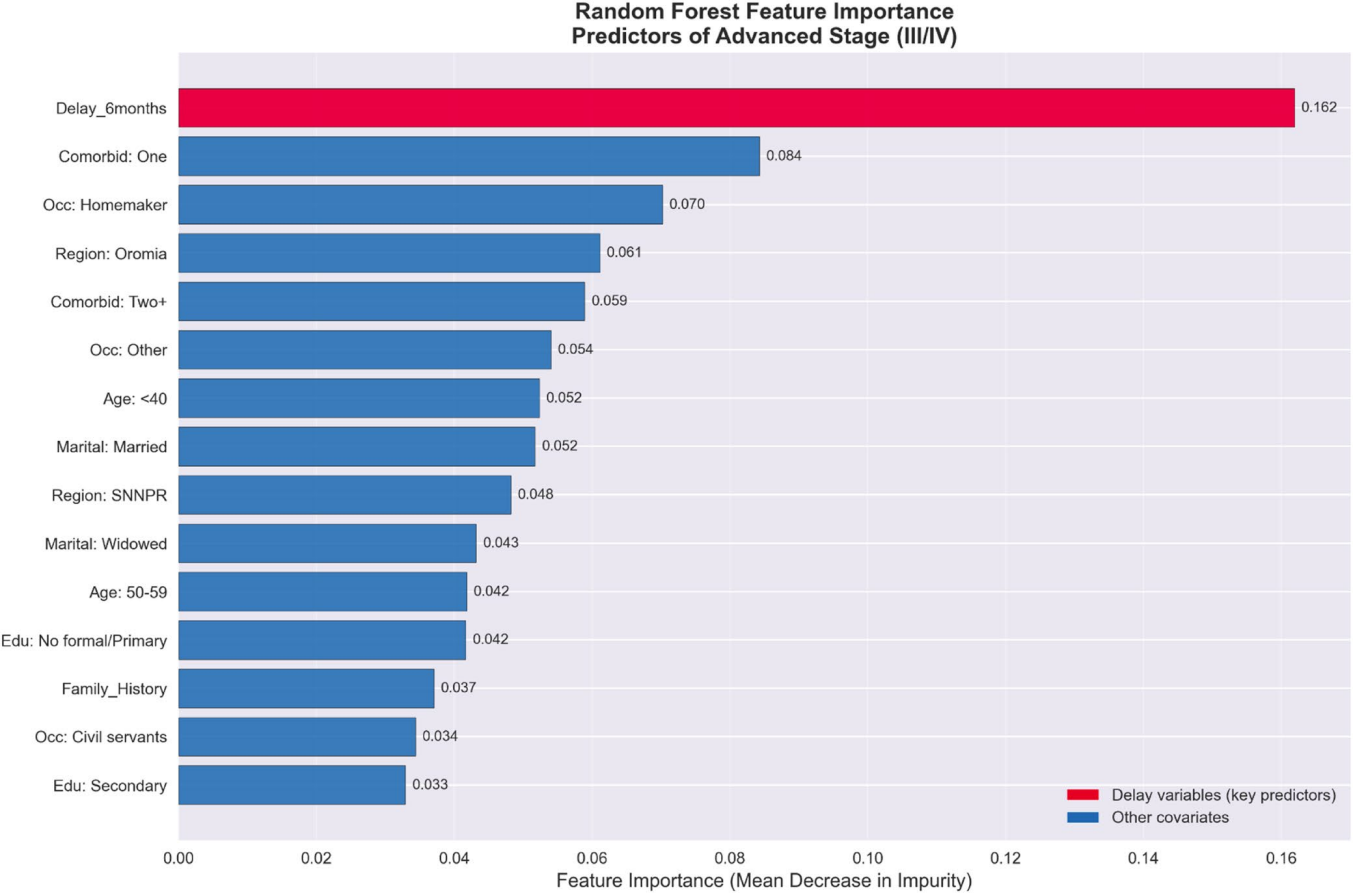
Random forest variable importance plot for predictors of advanced stage at presentation.

**FIGURE 4:**
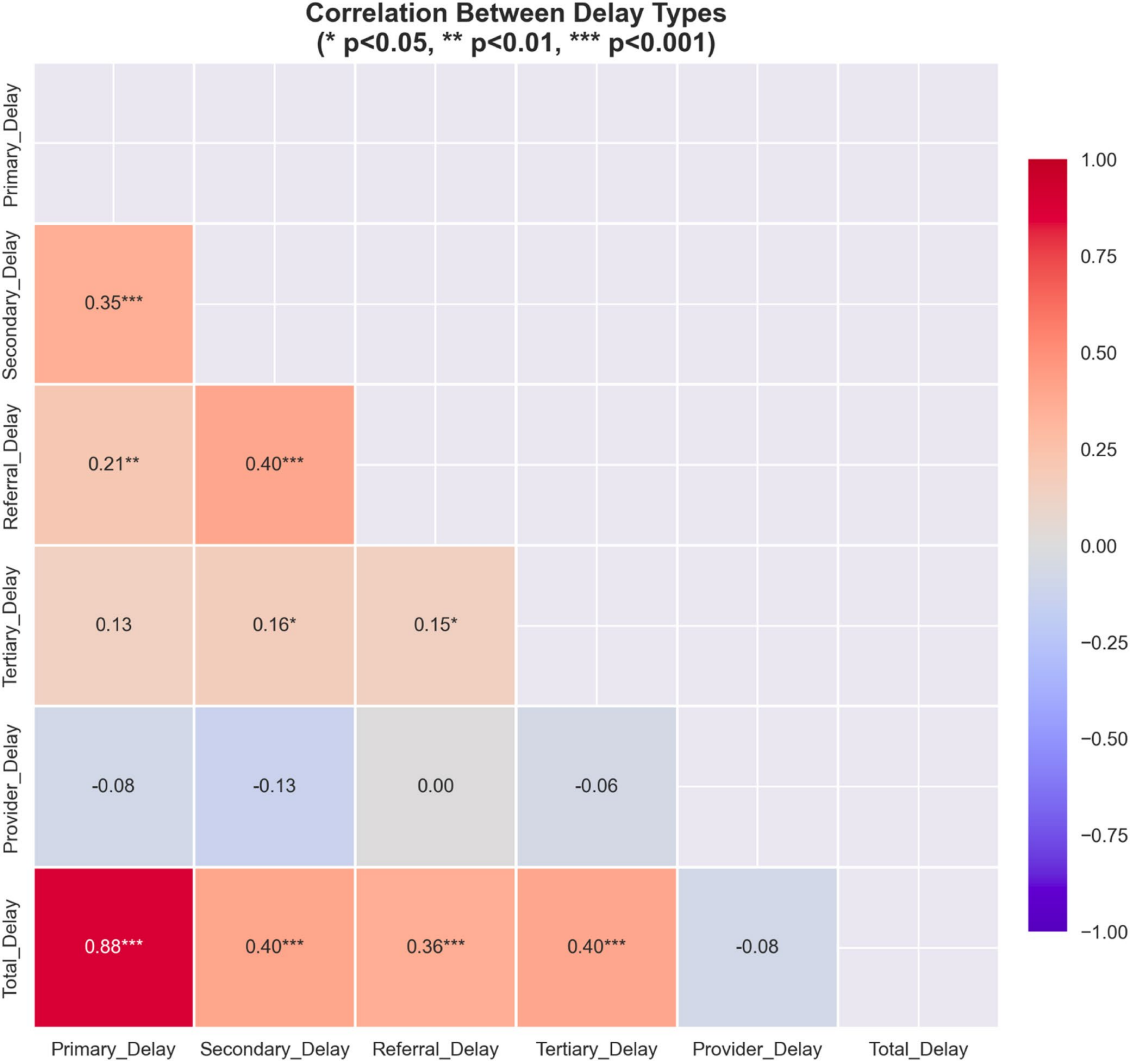
Cross‐correlation matrix: Delay components and total delay.

In multivariable logistic regression analysis, total delay ≥ 6 months was independently associated with late‐stage (III/IV) presentation. In the fully adjusted model, patients experiencing delay ≥ 6 months had 4.26‐fold higher odds of presenting with advanced stage (stage III and IV) compared to those with shorter delays (AOR 4.26, 95% CI: 2.07–8.77, *p* < 0.001). This association remained consistent across hierarchical models, with odds ratios ranging from 2.81 to 4.26 after sequential adjustment for demographic, socioeconomic, and clinical factors (Figure 2). Random forest analysis corroborated these findings, identifying delay ≥ 6 months as the most important predictor of advanced stage (importance score 0.162), with an overall model accuracy of 83.9%. When delay components were examined separately, primary delay showed a strong association (AOR per 1 SD: 2.48, 95% CI:: 1.46–4.22, *p* < 0.0001; RFI = 0.356) (Figure 3). Secondary delay also demonstrated a significant association (AOR per 1 SD: 2.61, 95% CI: 1.42–4.79, *p* = 0.002), though its random forest importance was lower (0.114). Correlation analysis revealed that primary delay is the definitive driver of the diagnostic timeline (*r* = 0.88, *p* < 0.001), accounting for over 77% of the total variance in delay. This extreme correlation confirms that the total duration of care is largely predetermined by the patient's initial decision‐making interval, with no significant compression of time occurring during the clinical pathway. The variance in TD is explained the least with provider delay among all the delay categories (*r* = −0.081) Table 2, Figures 4 and S2.

**TABLE 2:** Univariable and multivariable regression of delay types with stages of disease at the time of treatment.

Delay type	UOR (95% CI)^a^	MOR (per 1 SD)^b^	RFI^c^	Median (IQR) late stage	Median (IQR) early stage
Primary delay	1.10 (1.04–1.16)	**2.48 (1.46–4.22)**	**0.356**	4.0 (1.0–12.0)	2.0 (0.5–4.0)
Secondary delay	3.16 (1.53–6.56)	**2.61 (1.42–4.79)**	0.114	0.0 (0.0–1.0)	0.0 (0.0–0.0)
Referral delay	1.03 (0.94–1.12)	1.17 (0.72–1.94)	0.110	0.0 (0.0–0.4)	0.0 (0.0–0.2)
Provider delay	1.06 (0.99–1.13)	1.50 (0.93–2.41)	0.191	0.0 (0.0–2.0)	0.0 (0.0–1.0)
Tertiary delay	1.05 (0.94–1.18)	1.14 (0.83–1.56)	0.227	1.0 (0.5–2.0)	0.8 (0.5–2.0)

*Note:* Primary delay: *p*‐value 0.0004; Secondary delay: *p*‐value 0.0118 (in bold).

^a^ Univariable odds ratio (95% confidence interval).

^b^ Multivariable odds ratio (per 1 standard deviation).

^c^ Random forest importance.

**FIGURE 5:**
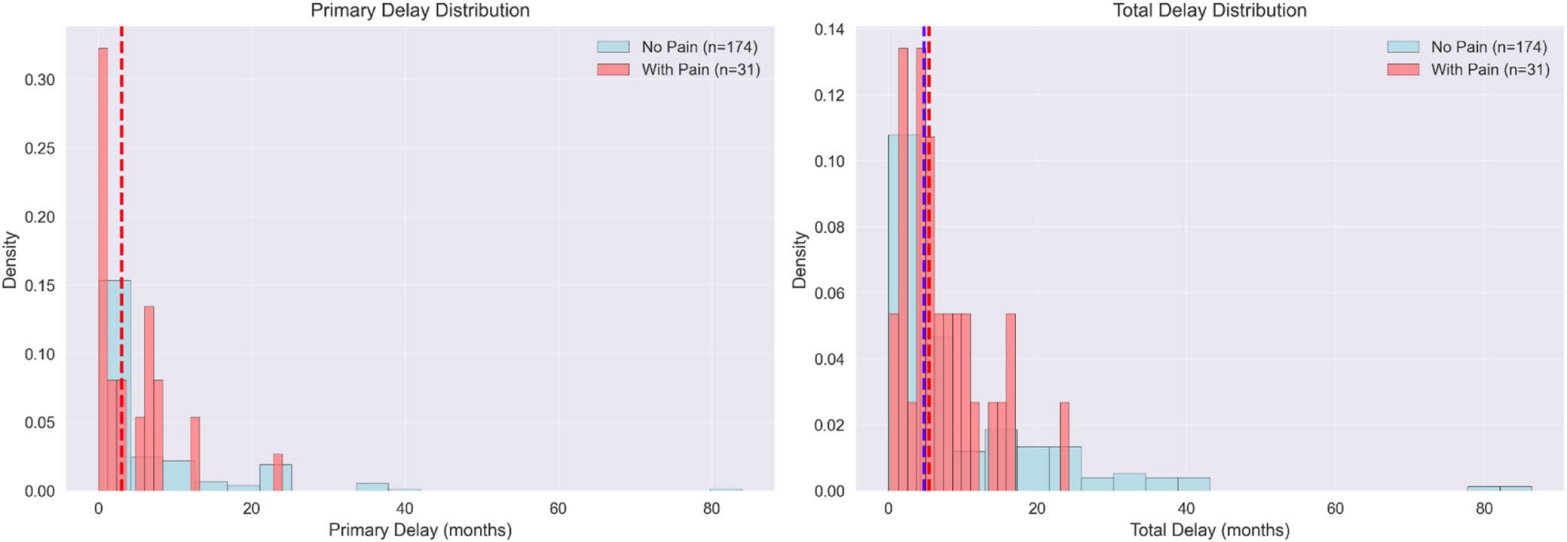
Primary and total delay distribution among patients with breast pain compared to those with no pain.

The association between absence of pain and delays in presentation at both the PD and TD levels was evaluated. Patients presenting with breast pain had a median PD of 3 months (mean 4.36 ± 5.06 months; range: 0–24 months), while those without breast pain also had a median PD of 3 months (mean 7.0 ± 10.46 months; range: 0–84 months). Regarding TD, the pain and no‐pain groups had median delays of 5.5 months (mean 7.24 ± 5.51 months) and 4.81 months (mean 9.94 ± 12.73 months), respectively. Mann–Whitney *U* tests revealed no significant differences between patients with and without breast pain in either PD (*p* = 0.473) or TD (*p* = 0.746). However, the broader dispersions of PD and TD in the absence of pain indicate that lack of pain may contribute to extreme delays in a minority of patients (Figure 5).

The association between education level and reported lack of awareness was examined. Lack of awareness was reported by 88.8% (*n* = 103) of patients with primary school education or less, 89.7% (*n* = 35) of those with secondary school education, and 76.0% (*n* = 19) of those with college education. No statistically significant association was observed between education level and lack of awareness (*χ* ^2^ = 3.3, *p* = 0.196).

During PD, 27.8% of patients (*n* = 56) reported using complementary and alternative medicine (CAM), of whom 23.3% (*n* = 47) used religious alternatives exclusively. During SD and TD, religious alternatives were utilized by 20.0% (*n* = 43) and 13.2% (*n* = 29) of patients, respectively. The median TD was 5.2 months among CAM users and 4.6 months among non‐users. Although the mean TD was numerically higher among CAM users (12.4 ± 14.9 months) compared with non‐users (8.45 ± 10.5 months), this difference did not reach statistical significance (Mann–Whitney *U* test, *p* = 0.073). Further stratified analyses demonstrated no significant association between CAM use and intermediate (3–6 months) or prolonged delays (> 6 months and > 12 months). Subgroup analysis by CAM type (religious, traditional, or combined modalities) similarly showed no statistically significant differences in TD compared with non‐users (Kruskal–Wallis test: *H* = 5.92, *p* = 0.052). Overall, CAM utilization was not significantly associated with TD in this cohort (Figure S3). Treatment modalities used for the patient cohort are presented in Figure S4.

## 4 Discussion

In this multicenter study of Ethiopian patients with breast cancer, prolonged diagnostic and treatment intervals were common and strongly associated with advanced‐stage presentation. More than half of patients experienced delays exceeding 6 months from symptom onset to treatment initiation, and total delay ≥ 6 months was independently associated with a more than fourfold increase in the odds of presenting with stage III–IV disease. Among the individual components of the diagnostic pathway, the primary delay emerged as the dominant contributor to overall delay, demonstrating both the strongest association with advanced stage and the highest variable importance in machine learning analysis. In contrast, provider‐ and system‐related intervals contributed comparatively less to the variance in total delay. Although absence of breast pain and complementary and alternative medicine use were frequently reported, neither was independently associated with prolonged total delay, suggesting that structural and awareness‐related factors may play a more substantial role than symptom characteristics or parallel care‐seeking behaviors. Together, these findings highlight the critical importance of early symptom recognition and timely first healthcare contact in reducing late‐stage presentation in this setting.

The high prevalence of advanced‐stage breast cancer in Ethiopia is primarily driven by prolonged diagnostic and treatment intervals rather than inherent biological aggressiveness [11]. This study found that a total delay of 6 months or more independently increases the odds of advanced‐stage (Stage III/IV) presentation by more than fourfold. This “stage‐shift” is statistically significant, as the hazard of progressing from early to locally advanced disease escalates from 3% at 30 days to 31% by 90 days [12]. Given that the median total delay in this cohort was 152 days (5.05 months), it is estimated that at least one‐third of these patients experienced a preventable progression to advanced disease during their diagnostic journey.

Within the Ethiopian context, several studies have documented the consequences of delayed breast cancer diagnosis. Gebremariam et al. examined the impact of delay on survival, reporting a significantly increased hazard ratio for mortality among patients with late presentations [13]. Similarly, a pooled analysis of 24 Ethiopian studies revealed that 65% of patients presented at an advanced stage, identifying the primary driver as the delay in initial healthcare contact, a combination of what we define as primary and secondary delays [14]. Furthermore, a review of 20 studies by Zewdie et al. found that advanced stage was significantly associated with delays in seeking care of > 3 months and health system delays of > 2 months [4]. Collectively, these studies corroborate our results, while our findings further strengthen and itemize specific delay scenarios and their individual time points.

The most critical finding of this study is the statistical and practical primacy of the “Primary Delay,” defined as the interval between the initial recognition of symptoms and the first contact with a healthcare provider. Quantitative analysis revealed that this stage accounted for more than 77% of the total variance in the overall time to treatment, indicating that the majority of diagnostic delay occurs before the patient even enters the healthcare system. This delay is predominantly driven by insufficient awareness of breast cancer symptoms, with 77.8% of patients reporting limited knowledge regarding the clinical significance of early warning signs. Furthermore, the analysis demonstrated that general education level did not significantly correlate with cancer‐specific health literacy, suggesting that formal education alone does not adequately prepare individuals to recognize the early manifestations of breast cancer. These findings stood in stark contrast to previous studies from low‐resource settings, which demonstrated greater delays and more advanced stages of presentation among cohorts with lower formal education [15, 16]. Our findings highlight a critical gap between general educational attainment and disease‐specific awareness, emphasizing the need for targeted public health interventions and symptom‐awareness campaigns aimed specifically at improving recognition of early breast cancer signs and encouraging prompt medical evaluation.

Although 27.8% of patients in this cohort reported using complementary and alternative medicine (CAM), such as holy water, its use was not statistically associated with longer total delays. Subcomponent analysis of traditional, religious, and combined CAM modalities similarly showed no significant increase in the interval between symptom recognition and presentation to a healthcare provider. Previous studies have examined the role of CAM in breast cancer care from different perspectives. For example, Ayoade et al. reported that CAM use was associated with lower utilization of evidence‐based treatments such as radiotherapy and endocrine therapy, as well as poorer survival outcomes [17]. Furthermore, studies from Malaysia and Bangladesh have shown that reliance on traditional or alternative therapies may contribute to significantly longer delays in presentation and diagnosis [18, 19]. Conversely, other investigations suggest that spiritually based practices do not necessarily adversely affect clinical outcomes; for instance, Hulett et al. found that spiritual coping strategies were not associated with worse treatment outcomes among breast cancer patients [20, 21]. The heterogeneous findings across studies suggest that the influence of CAM on breast cancer care is context dependent and may vary according to cultural practices and patterns of healthcare utilization. In our cohort, CAM may function primarily as complementary coping mechanisms rather than substitutes for formal medical care, which could explain the lack of association with delay. Furthermore, because the primary objective of this study was not to evaluate the impact of CAM, the study may not have been adequately powered to detect a statistically significant association between CAM use and delays in presentation.

Several limitations to the interpretation of these findings should be acknowledged. First, because recruitment was conducted at tertiary referral centers, the cohort is inherently subject to selection and survivor bias. Women who died before reaching a hospital, as well as those in rural areas who never accessed the referral system, were not captured in the study population; consequently, the magnitude of delays observed here may underestimate the true burden present in the broader community. Second, reconstruction of the diagnostic pathway relied on retrospective patient recall of the timing of symptom onset and subsequent healthcare encounters. Such recall‐based estimates are inherently susceptible to imprecision and recall bias, which may have affected the accuracy of the reported time intervals.

In conclusion, achieving the World Health Organization's target of diagnosis within 60 days requires particular attention to the early stages of the breast cancer care pathway in Ethiopia [22]. The findings of this study highlight that the majority of delays occur during the pre‐clinical phase, before patients first engage with the healthcare system. In particular, the substantial contribution of primary delay suggests that improving community awareness and early recognition of breast cancer symptoms may represent a critical area for intervention. Expanding public education efforts and strengthening community‐level engagement could help promote earlier health‐seeking behavior and reduce the interval between symptom recognition and initial medical consultation.

## Author Contributions

**Meklit Solomon Gebremariam:** conceptualization, methodology, writing – review and editing. **Segni Kejela:** conceptualization, methodology, software, formal analysis, supervision, visualization, project administration, writing – original draft. **Yeabtsega Amlaku Asres:** data curation, investigation, validation, formal analysis. **Erko Chala Beyene:** methodology, data curation, writing – review and editing. **Tsegab Alemayehu Bukate:** data curation, investigation, validation, formal analysis. **Misgana Muluneh Ojamo:** data curation, investigation, validation, formal analysis. **Kalsidagn Girma Asfaw:** writing – review and editing, formal analysis. **Meti Temesgen Negassa:** methodology, data curation, writing – original draft. **Abenezer Tarekegne Legesse:** data curation, writing – review and editing.

## Funding

The authors have nothing to report.

## Ethics Statement

Ethical approval was obtained from the Institutional Review Board of the Department of Surgery at Addis Ababa University College of Health Sciences and Saint Paul's Millenium Medical College through an expedited review process for a residency graduation thesis Written informed consent was obtained from all participants prior to enrollment. All data were anonymized and handled in accordance with institutional and national ethical guidelines.

## Consent

The authors have nothing to report.

## Conflicts of Interest

The authors declare no conflicts of interest.

## Supporting information


**Table S1:** Baseline socio‐demographic characteristics of the study population.
**Table S2:** Clinicopathologic factors of the respondents.
**Figure S1:** Flowchart of inclusion and exclusion criteria.
**Figure S2:** Pairwise Pearson correlation coefficients between individual delay intervals (primary, secondary, referral, tertiary, provider) and total delay across the breast cancer care continuum.
**Figure S3:** Comparison of total delay distribution between CAM users and non‐users.
**Figure S4:** Modalities of management (a) proportion of patients with curative, palliative and no surgery (b) type of surgery performed (c) chemotherapy.

## Data Availability

The data that support the findings of this study are available on request from the corresponding author. The data are not publicly available due to privacy or ethical restrictions.

## References

[1] D. D. Tang , Z. J. Ye , W. W. Liu , et al., “Survival Feature and Trend of Female Breast Cancer: A Comprehensive Review of Survival Analysis From Cancer Registration Data,” Breast 79 (2025): 103862, 10.1016/j.breast.2024.103862. 39701013 PMC11722932

[2] T. Shimels , A. I. Billal , A. M. Fentie , T. G. Fenta , and T. Gebre‐Mariam , “Perception, Awareness, Knowledge, and Attitude (PAKA) Towards Breast Cancer in Ethiopia: A Systematic Review and Meta‐Analysis,” Journal of Primary Care & Community Health 16 (2025): 21501319251396797, 10.1177/21501319251396797. PMC12647559 41288190

[3] A. M. Filho , M. Laversanne , J. Ferlay , et al., “The GLOBOCAN 2022 Cancer Estimates: Data Sources, Methods, and a Snapshot of the Cancer Burden Worldwide,” International Journal of Cancer 156, no. 7 (2025): 1336–1346, 10.1002/ijc.35278. 39688499

[4] A. Zewdie , T. D. Kassie , T. F. Anagaw , et al., “Advanced‐Stage Breast Cancer Diagnosis and Its Determinants in Ethiopia: A Systematic Review and Meta‐Analysis,” BMC Women's Health 24, no. 1 (2024): 284, 10.1186/s12905-024-03133-9. 38734607 PMC11088059

[5] A. Gadgil , R. Srinivasan , S. Kantharia , and P. Basu , “Disrupting the Status Quo in Late Diagnosis of Breast Cancers in the Resource Limited Settings,” Medical Research Archives 11, no. 11 (2023), 10.18103/mra.v11i11.4627.

[6] A. A. Kibret , H. Jiang , H. Yang , and C. Liu , “Patient Journey and Timeliness of Care for Patients With Breast Cancer in Africa: A Scoping Review Protocol,” BMJ Open 14, no. 9 (2024): e081256, 10.1136/bmjopen-2023-081256. PMC11381707 39242165

[7] P. Boucheron , A. Zietsman , A. Anele , et al., “Measuring the WHO Global Breast Cancer Initiative Pillars' Key Performance Indicators in Sub‐Saharan Africa: Experience in the African Breast Cancer‐Disparities in Outcomes Hospital‐Based Cohort Study,” EClinicalMedicine 81 (2025): 103104, 10.1016/j.eclinm.2025.103104. 40034567 PMC11872636

[8] M. A. Richards , A. M. Westcombe , S. B. Love , P. Littlejohns , and A. J. Ramirez , “Influence of Delay on Survival in Patients With Breast Cancer: A Systematic Review,” Lancet 353, no. 9159 (1999): 1119–1126, 10.1016/s0140-6736(99)02143-1. 10209974

[9] D. Weller , P. Vedsted , G. Rubin , et al., “The Aarhus Statement: Improving Design and Reporting of Studies on Early Cancer Diagnosis,” British Journal of Cancer 106, no. 7 (2012): 1262–1267, 10.1038/bjc.2012.68. 22415239 PMC3314787

[10] V. Actis Danna , C. Bedwell , S. Wakasiaka , and T. Lavender , “Utility of the Three‐Delays Model and Its Potential for Supporting a Solution‐Based Approach to Accessing Intrapartum Care in Low‐ and Middle‐Income Countries. A Qualitative Evidence Synthesis,” Global Health Action 13, no. 1 (2020): 1819052, 10.1080/16549716.2020.1819052. 33040697 PMC7580724

[11] S. Zhou , “Bridging Knowledge Gaps in Breast Cancer Prevention: Insights From Ethiopia,” World Journal of Clinical Oncology 16, no. 7 (2025): 106687, 10.5306/wjco.v16.i7.106687. 40741190 PMC12304897

[12] O. Agodirin , S. Olatoke , G. Rahman , et al., “Presentation Intervals and the Impact of Delay on Breast Cancer Progression in a Black African Population,” BMC Public Health 20, no. 1 (2020): 962, 10.1186/s12889-020-09074-w. 32560711 PMC7304119

[13] A. Gebremariam , A. Addissie , A. Worku , et al., “Association of Delay in Breast Cancer Diagnosis With Survival in Addis Ababa, Ethiopia: A Prospective Cohort Study,” JCO Global Oncology 9 (2023): e2300148, 10.1200/GO.23.00148. 37992269 PMC10681531

[14] H. Geremew , E. B. Golla , M. B. Simegn , et al., “Late‐Stage Diagnosis: The Driving Force Behind High Breast Cancer Mortality in Ethiopia: A Systematic Review and Meta‐Analysis,” PLoS One 19, no. 7 (2024): e0307283, 10.1371/journal.pone.0307283. 39028722 PMC11259299

[15] N. A. Ibrahim and M. A. Oludara , “Socio‐Demographic Factors and Reasons Associated With Delay in Breast Cancer Presentation: A Study in Nigerian Women,” Breast 21, no. 3 (2012): 416–418, 10.1016/j.breast.2012.02.006. 22381153

[16] Y. Liu , J. Zhang , R. Huang , et al., “Influence of Occupation and Education Level on Breast Cancer Stage at Diagnosis, and Treatment Options in China: A Nationwide, Multicenter 10‐Year Epidemiological Study,” Medicine (Baltimore) 96, no. 15 (2017): e6641, 10.1097/MD.0000000000006641. 28403116 PMC5403113

[17] O. F. Ayoade , G. Caturegli , M. E. Canavan , B. J. Resio , E. R. Berger , and D. J. Boffa , “Use of Complementary and Alternative Medicine in the Management of Breast Cancer,” JAMA Network Open 9, no. 3 (2026): e260337, 10.1001/jamanetworkopen.2026.0337. 41770560 PMC12954545

[18] N. M. Mohd Mujar , M. Dahlui , N. A. Emran , et al., “Complementary and Alternative Medicine (CAM) Use and Delays in Presentation and Diagnosis of Breast Cancer Patients in Public Hospitals in Malaysia,” PLoS One 12, no. 4 (2017): e0176394, 10.1371/journal.pone.0176394. 28448541 PMC5407802

[19] K. Akhtar , K. Akhtar , and M. M. Rahman , “Use of Alternative Medicine Is Delaying Health‐Seeking Behavior by Bangladeshi Breast Cancer Patients,” European Journal of Breast Health 14, no. 3 (2018): 166–172, 10.5152/ejbh.2018.3929. 30123883 PMC6092151

[20] J. M. Hulett and J. M. Armer , “A Systematic Review of Spiritually Based Interventions and Psychoneuroimmunological Outcomes in Breast Cancer Survivorship,” Integrative Cancer Therapies 15, no. 4 (2016): 405–423, 10.1177/1534735416636222. 27151592 PMC5125023

[21] J. M. Hulett , J. M. Armer , E. Leary , et al., “Spirituality, and Salivary Cortisol in Breast Cancer Survivorship: A Pilot Study,” Cancer Nursing 41, no. 2 (2018): 166–175, 10.1097/NCC.0000000000000471. 28151830 PMC5540803

[22] S. K. Ong , R. Haruyama , C. H. Yip , et al., “Feasibility of Monitoring Global Breast Cancer Initiative Framework Key Performance Indicators in 21 Asian National Cancer Centers Alliance Member Countries,” EClinicalMedicine 67 (2023): 102365, 10.1016/j.eclinm.2023.102365. 38125964 PMC10731600

