# Supplementary material for: Assessing Delay Patterns in Diagnosis and Care Among Breast Cancer Patients in Ethiopia
Source: Cancer Rep (Hoboken). 2026 May 31;9(6):e70593. doi: 10.1002/cnr2.70593 (PMC13239925; doi:10.1002/cnr2.70593)

**Supplemental File**

**Supplemental Table 1. Baseline socio-demographic characteristics of the study population**

| **Category** | **Subcategory** | **Number** | **Percentage** |
| --- | --- | --- | --- |
| **Sex** | Female | 196 | 95.6 |
|  | Male | 9 | 4.4 |
| **Region of residence** | Addis Ababa | 114 | 55.1 |
|  | Oromia | 51 | 24.6 |
|  | Amhara | 17 | 8.2 |
|  | SNNRP* | 16 | 7.7 |
|  | Tigrai | 6 | 2.9 |
| **‍** | Other | 4 | 1.8 |
| **Marital status** | Married | 158 | 77.1 |
|  | Widowed | 20 | 9.7 |
|  | Single | 20 | 9.7 |
|  | Divorced | 7 | 3.4 |
| **Occupation** | Homemaker | 99 | 48.3 |
|  | Civil servants | 34 | 16.6 |
|  | Business owner | 28 | 13.7 |
|  | Retired | 13 | 6.3 |
|  | Unemployed | 13 | 6.3 |
|  | Student | 1 | 0.5 |
|  | Other | 17 | 8.3 |
| **Educational status** | Finished College Education | 30 | 14.8 |
|  | Completed Secondary School | 40 | 19.8 |
|  | Completed Primary School | 55 | 27.2 |
|  | Did Not Complete Primary School | 77 | 38.1 |

* South Nations Nationalities and Peoples Region

**Supplemental Table 2: clinicopathologic factors of the respondents**

| **Category** | **Subcategory** | **Number** | **Percentage** |
| --- | --- | --- | --- |
| **Comorbidities** | Hypertension | 39 | 18.8 |
|  | Diabetes | 17 | 8.2 |
|  | Neurologic disorder | 5 | 2.4 |
|  | Cardiac illness | 5 | 2.4 |
|  | HIV | 4 | 2 |
|  | None | 135 | 65.9 |
| **Family history** | No | 173 | 84.5 |
|  | Yes | 32 | 15.5 |
| **Presenting clinical symptom** | Breast lump | 180 | 89.1 |
|  | Breast pain | 29 | 14.4 |
|  | Axillary lump | 20 | 9.9 |
|  | Nipple discharge | 16 | 7.9 |
|  | Nipple change | 11 | 5.4 |
|  | Skin ulceration | 9 | 4.4 |
|  | Other | 9 | 4.4 |
| **Side of breast involved** | Left | 114 | 55.6 |
|  | Right | 87 | 42.4 |
|  | Bilateral | 4 | 2 |
| **Stage at definitive treatment time** | Stage I | 13 | 6.3 |
|  | Stage II | 62 | 30.2 |
|  | Stage III | 96 | 46.8 |
|  | Stage IV | 34 | 16.6 |
| **Histologic type** | Ductal carcinoma | 163 | 79.5 |
|  | Lobular carcinoma | 9 | 4.4 |
|  | Undifferentiated | 12 | 5.8 |
|  | Other histologies | 11 | 5.4 |
|  | Not mentioned | 10 | 4.9 |
| **Grade of tumor** | Grade I | 13 | 6.3 |
|  | Grade II | 44 | 21.5 |
|  | Grade III | 48 | 23.4 |
|  | Unspecified | 100 | 48.8 |

**Supplemental Figure 1: Flowchart of inclusion and exclusion criteria**





**Supplemental Figure 2: Pairwise Pearson Correlation Coefficients Between Individual Delay Intervals (Primary, Secondary, Referral, Tertiary, Provider) and Total Delay Across the Breast Cancer Care Continuum**


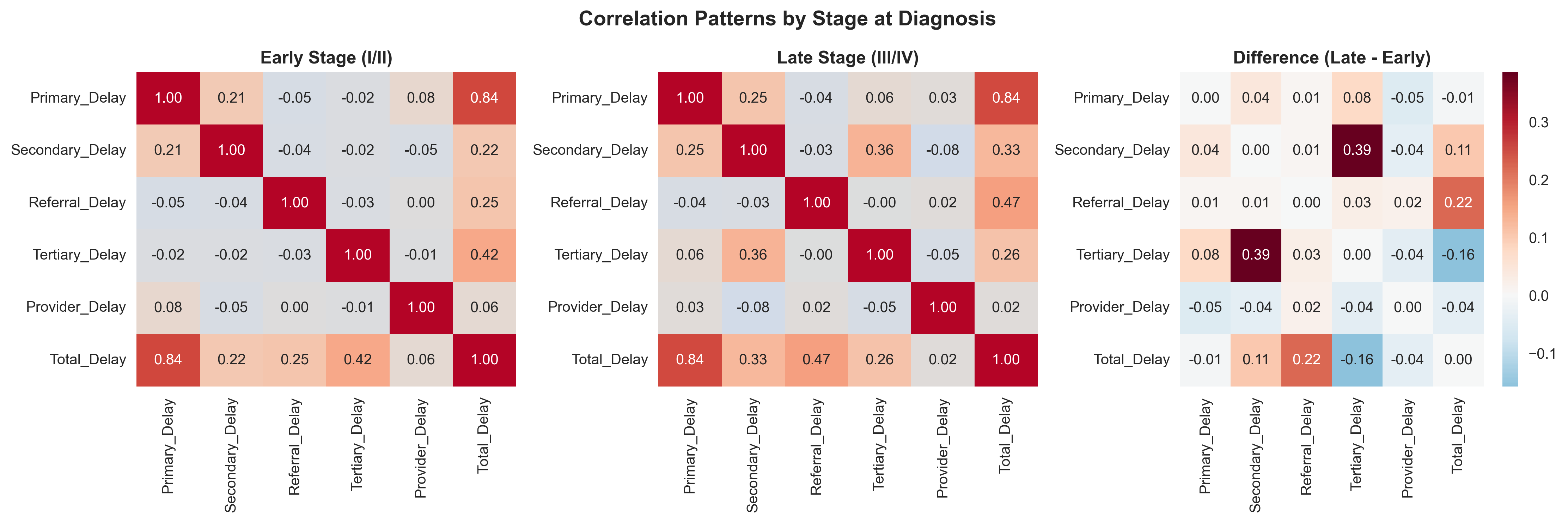


**Supplemental Figure 3: Comparison of Total Delay Distribution Between CAM Users and Non-Users**


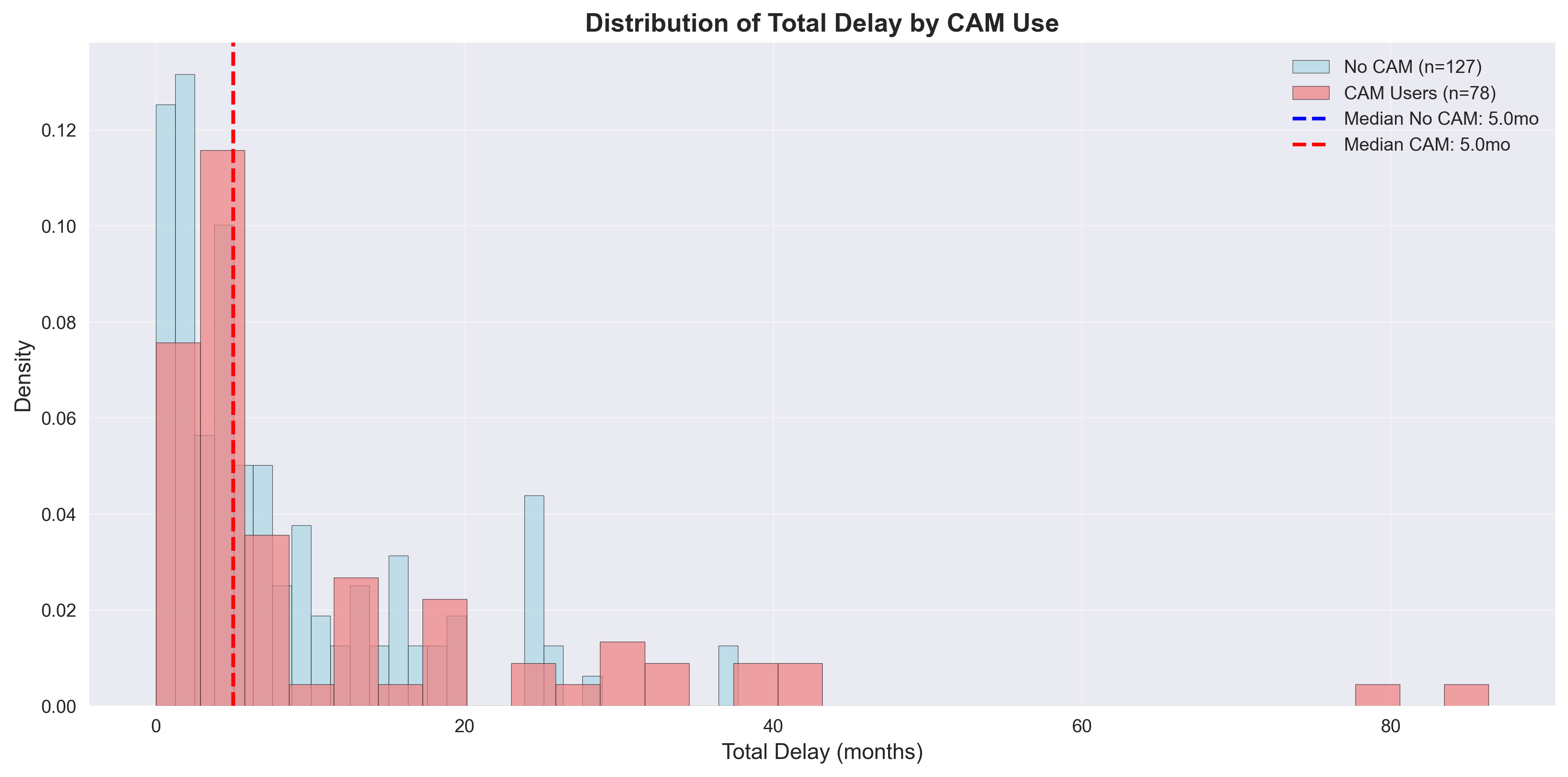


**Supplemental Figure 4: modalities of management (a) proportion of patients with curative, palliative and no surgery (b) type of surgery performed (c) chemotherapy**


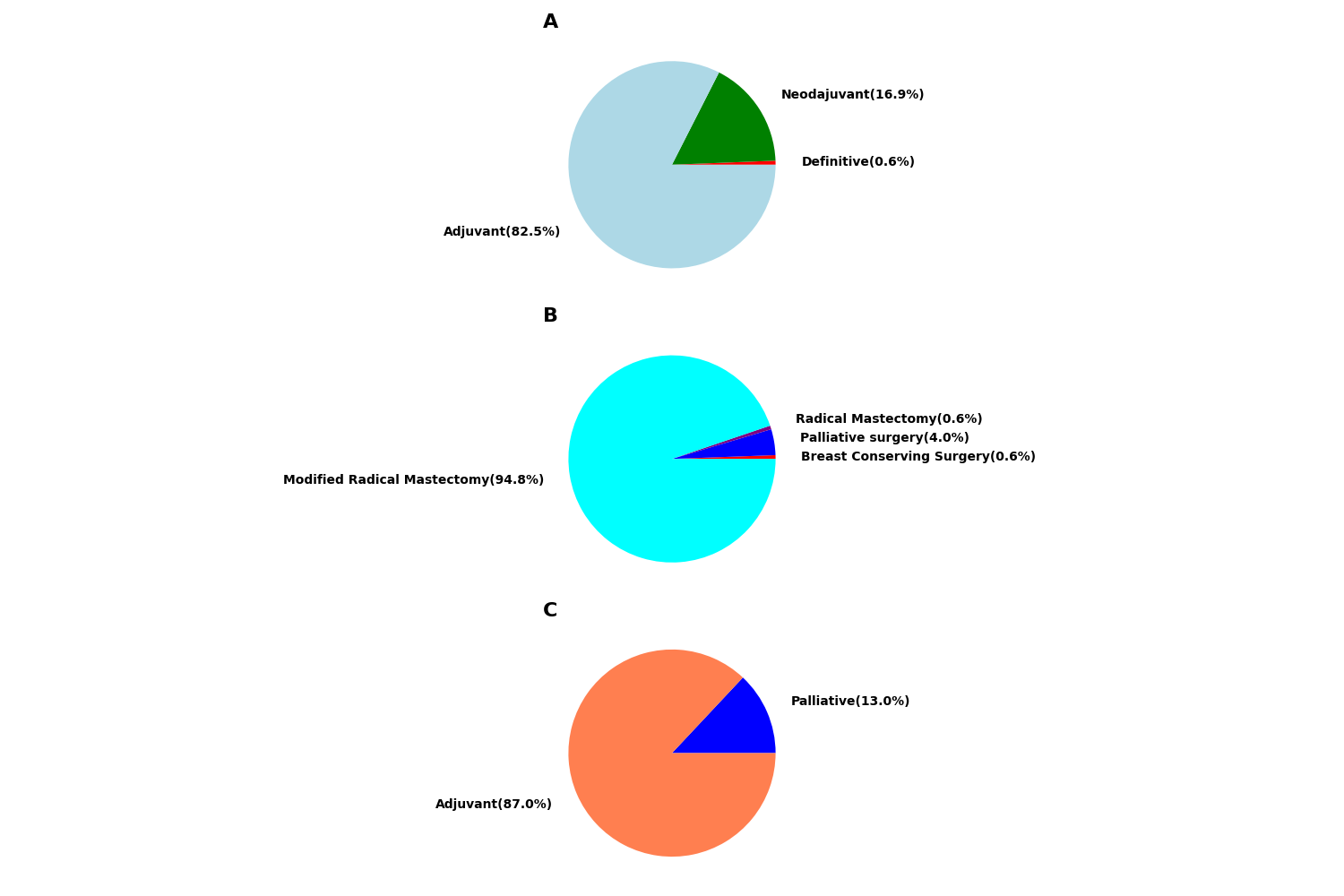

Supplement: Supplementary file 1 — Table S1: Baseline socio‐demographic characteristics of the study population. Table S2: Clinicopathologic factors of the respondents. Figure S1: Flowchart of inclusion and exclusion criteria. Figure S2: Pairwise Pearson correlation coefficients between individual delay intervals (primary, secondary, referral, tertiary, provider) and total delay across the breast cancer care continuum. Figure S3: Comparison of total delay distribution between CAM users and non‐users. Figure S4: Modalities of management (a) proportion of patients with curative, palliative and no surgery (b) type of surgery performed (c) chemotherapy. [file CNR2-9-e70593-s001.docx]
